# ﻿Discovery of Laophontidae (Copepoda, Harpacticoida) from marine plastic debris: *Pseudonychocamptus setadefectus* sp. nov. and *Heterolaophonte discophora* (Willey, 1929)

**DOI:** 10.3897/zookeys.1251.160858

**Published:** 2025-09-15

**Authors:** Kyuhee Cho, Jong Guk Kim, Jimin Lee

**Affiliations:** 1 Ocean Climate Response & Ecosystem Research Department, Korea Institute of Ocean Science & Technology, Busan 49111, Republic of Korea Ocean Climate Response & Ecosystem Research Department, Korea Institute of Ocean Science & Technology Busan Republic of Korea; 2 Division of Zoology, Honam National Institute of Biological Resources, Mokpo 58762, Republic of Korea Division of Zoology, Honam National Institute of Biological Resources Mokpo Republic of Korea

**Keywords:** Biodiversity, Crustacea, *
Heterolaophonte
*, meiofauna, *
Pseudonychocamptus
*, taxonomy

## Abstract

Two species belonging to the family Laophontidae Scott T., 1905 were identified among harpacticoid copepods collected from marine plastic debris (MPD) stranded along the Korean coastline. These species were assigned to the genera *Pseudonychocamptus* Lang, 1944 and *Heterolaophonte* Lang, 1948. *Pseudonychocamptus setadefectus***sp. nov.** conforms to the generic diagnosis, displaying sexual dimorphism in the armature of the swimming legs. It is distinguished from its six congeners by possessing only two endites on the maxillary syncoxa, notably lacking the proximal endite bearing the seta that is present in all other known species within the genus. Although most morphologically similar to *P. colomboi* Ceccherelli, 1988, the new species differs in several key characteristics, including the number of setae on the maxillary endopod, the relative length ratios of setae on the female P5 exopod, the presence of an inner seta on the male P5 exopod, and the asymmetry of the male P6. The second species, *Heterolaophonte discophora* (Willey, 1929), was previously reported from the Atlantic coast of Canada and the Pacific coasts of the USA and Japan. The Korean specimens closely correspond to these previous records, yet exhibit intraspecific variation, particularly in the setal count on the female P3 enp-2. Additional morphological differences were also observed, including variations in the segmentation of the male P3 endopod and in the number of setae on the maxillary endopod. Based on the sexually dimorphic traits observed in males, we propose the subdivision of the genus *Heterolaophonte* into four distinct species groups. The discovery of *P. setadefectus***sp. nov.** and *H. discophora* on anthropogenic MPD underscores the potential role of such substrates as microhabitats for benthic harpacticoid copepods.

## ﻿Introduction

The family Laophontidae Scott T., 1905 represents one of the most diverse groups of benthic harpacticoid copepods, encompassing two subfamilies, Esolinae Huys & Lee, 2000 and Laophontinae Scott T., 1905, and 77 genera with 353 species (Walter and Boxshall 2025). Members of this family exhibit remarkable ecological adaptability, inhabiting a wide range of aquatic environments, including freshwater, brackish, and marine ecosystems, from the intertidal zone to the deep sea. Although laophontids are commonly found in intertidal and subtidal sediments, many species are also closely associated with macroalgae, seagrasses, and various invertebrates, such as cnidarians, mollusks, bryozoans, crustaceans, and echinoderms (Hicks 1977a, b; Ho and Perkins 1977; Gotto 1979; Kim 1991; Fiers 1992; Huys et al. 1996; Cottarelli et al. 2006; Huys 2016; Yeom et al. 2018). In addition, laophontid copepods have been documented from a variety of other substrates, including submerged woods, coral, sponges, and oysters (Fiers and Rutledge 1990; Gheerardyn et al. 2006, 2007; Kim 2013). This ecological flexibility has facilitated their broad substrate range and contributed to their widespread geographical distribution.

Although recent taxonomic revisions [e.g., Huys and Lee 1999] have enhanced the morphological coherence of the family Laophontidae, the phylogenetic relationships among its constituent genera remain largely unresolved (Huys and Lee 2000). Recent molecular studies have provided strong support for the monophyly of certain genera, such as *Quinquelaophonte* (Kim and Lee 2023; Hou et al. 2025), but have yet to confirm the monophyly of the family as a whole. Many species were originally described with limited diagnostic detail, particularly in earlier literature, and frequently lack descriptions of male morphology, which is critical for accurate taxonomic resolution in harpacticoid copepods (Huys and Lee 2018; Gómez and Nazari 2021; Song et al. 2023). To clarify the relationships within the family, a comprehensive re-evaluation of generic definitions that integrates contemporary morphological data with molecular evidence is urgently needed.

Within the Korean fauna, up to 38 species belonging to 17 genera of the family Laophontidae have been reported (Kim 2013, 2014; NIBR 2025). However, only 15 species across nine genera have been formally described and documented through peer-reviewed taxonomic studies. These include: *Afrolaophonte koreana* Karanovic, 2022; *Echinolaophonte musa* Song, Lee, Kim, George & Khim, 2023; *Folioquinpes pseudomangalis* Huys & Lee, 2018; *Jejulaophonte hyeopjaeensis* Back & Lee, 2014; *Microchelonia koreensis* (Kim, 1991); *Onychocamptus bengalensis* (Sewell, 1934); *O. mohammed* (Blanchard & Richard, 1891); *O. vitiospinulosa* (Shen & Tai, 1963); *Paralaophonte congenera* (Sars G.O., 1908); *P. naroensis* Karanovic, 2023; *Philippiphonte aspidosoma* Huys & Lee, 2018; *Quinquelaophonte enormis* Kim, Nam & Lee, 2020; *Q. koreana* Lee, 2003; *Q. sominer* Kim & Lee, 2023; and *Strictlaophonte hupoensis* Oh, Kim & Lee, 2024 (MABIK 2025).

Conversely, earlier records of known 23 species in Korean fauna (e.g., Kim 2013, 2014) were based on limited morphological descriptions or photographic images, lacking the diagnostic detail necessary for formal taxonomic identification. This suggests that a substantial portion of the laophontid fauna in Korea remains taxonomically unverified. Subsequent studies investigating the Korean harpacticoid fauna have revealed that some of these records were either misidentifications or represented incompletely described species (Huys and Lee 2018; Song et al. 2023). Notably, specimens previously assigned to *Echinolaophonte mirabilis* (Gurney, 1927) and *Folioquinpes mangalis* Fiers & Rutledge, 1990 were later reclassified as new species, *E. musa* and *F. pseudomangalis*, respectively (Huys and Lee 2018; Song et al. 2023). These findings underscore the urgent need for a comprehensive reassessment of previously reported laophontid copepods from Korea to clarify species identities and update the national faunal inventory based on rigorous taxonomic standards.

Recent studies have identified marine plastic debris (MPD) as both a rafting vector for non-indigenous species and a novel ecological niche for a variety of marine invertebrates (Audrézet et al. 2021). During a survey of meiofaunal communities inhabiting anthropogenic debris stranded along the Korean coastline, two laophontid harpacticoid copepods were collected: one representing a new species belonging to the genus *Pseudonychocamptus* Lang, 1944, and the other a species of the genus *Heterolaophonte* Lang, 1948, which has not been previously documented in the peer-reviewed literature from Korea. In this study, we provide detailed morphological (re)descriptions of both sexes of these two species.

## ﻿Materials and methods

Samples were collected from various types of floating MPD at Gohado, Naechi, and Jeju Island, Republic of Korea. Additional specimens of *Heterolaophonte* were obtained by washing macroalgae collected from Masan-ri, Pohang. Copepods were extracted by rinsing the surfaces of debris with tap water using a compressed air sprayer. The dislodged organisms were filtered through a 60-μm mesh hand net and fixed in 90% ethanol at the sampling site. In the laboratory, laophontid harpacticoids were sorted from the preserved material under a stereomicroscope (M165 C; Leica Microsystems, Wetzlar, Germany). Specimens were dissected and temporarily mounted in lactic acid on reverse slides for microscopic examination. Morphometric measurements and illustrations were performed using a differential interference contrast light microscope (BX51; Olympus, Tokyo, Japan) equipped with a camera lucida. All scale bars in the figures are indicated in micrometers.

The dissected body parts were subsequently transferred to and permanently mounted in a lactophenol:glycerin solution (1:5) on H-S slides (Double slide plate, BSDS-011R; Biosolution, Republic of Korea) (cf. Shirayama et al. 1993), and sealed with transparent nail-varnish. Specimens of *Pseudonychocamptus* and *Heterolaophonte* were deposited in the Marine Biodiversity Institute of Korea (MABIK), Seocheon, Republic of Korea, and the Marine Interstitial fauna Resources Bank (MInRB) at KIOST, Busan, Republic of Korea. Accession numbers are provided in parentheses alongside each specimen.

**Figure 1..**
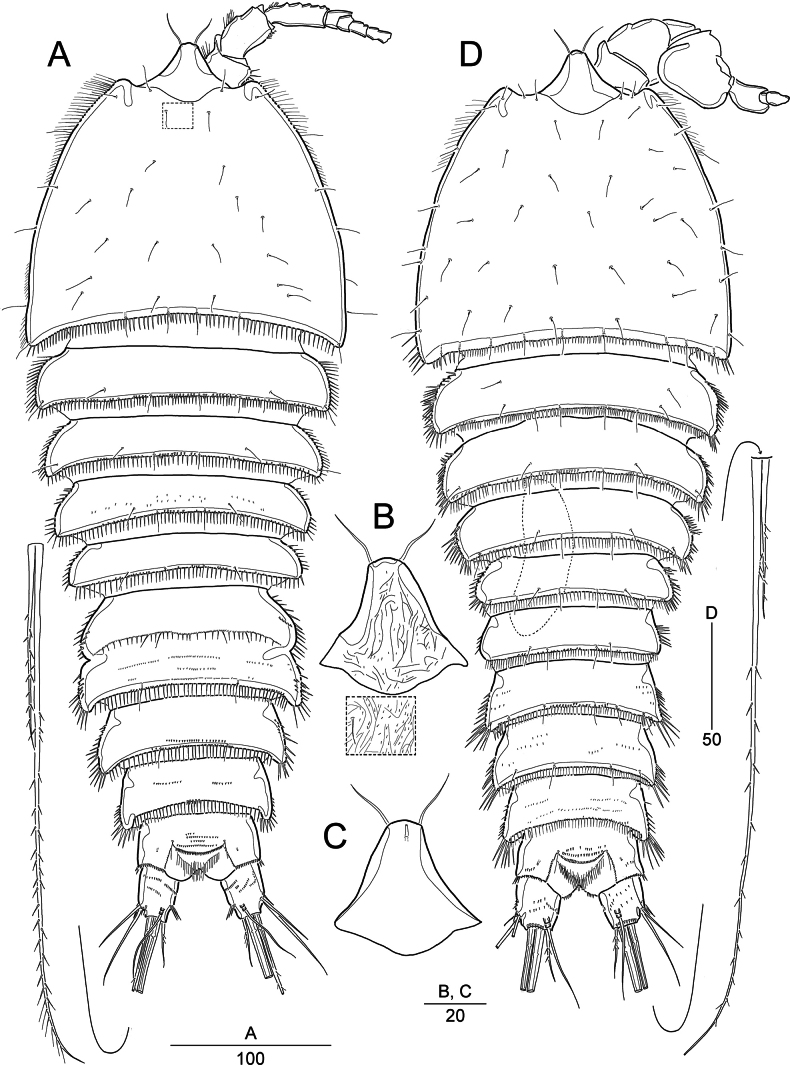
*Pseudonychocamptus setadefectus* sp. nov. Holotype (MABIK CR00258872), female: A. Habitus, dorsal; B. Rostrum, dorsal, and inset showing enlarged surface ornamentation on cephalothorax; C. Rostrum, ventral; Paratype (MABIK CR00258873), male: D. Habitus, dorsal. Scale bars are in μm.

The morphological terminology used in the text and figures follows Huys et al. (1996). The abbreviations used throughout the manuscript are as follows: **acro**, acrothek composed of one aesthetasc and two setae (fused basally); **ae**, aesthetasc; **enp**, endopod; **exp**, exopod; **exp(enp)-1 (-2, -3)**, proximal (middle, distal) segment of a ramus; and **P1–P6**, first to sixth thoracopod.

## ﻿Results

**Order Harpacticoida Sars, 1903**

**Family Laophontidae Scott T., 1905**

**Subfamily Laophontinae Scott T., 1905**

**Genus *Pseudonychocamptus* Lang, 1944**

### ﻿ Pseudonychocamptus setadefectus sp. nov.

Taxon classificationAnimaliaHarpacticoidaLaophontidae

05CAE939-04CB-55D2-BF28-0C5C7271FCD9

https://zoobank.org/FA68B5AD-14F1-4F30-A36B-254722A147CA

Figs 1, 2, 3, 4, 5

#### Type locality.

Gohado (34°45'56.04"N, 126°22'22.38"E), Mokpo, Jeollanam-do, Republic of Korea.

#### Type material.

***Holotype*.** • 1♀ (MABIK CR00258872), preserved in a vial with 95% ethanol, collected from washings of marine plastic debris (a gunny sack) at the type locality on 14 March 2022 by GH Han. ***Paratypes*.** • 2♀♀ (MABIK CR00258874–00258875), each dissected and mounted on 2 H-S slides; • 2♀♀ (MinRB-Hr104-L001), preserved in a vial with 95% ethanol; • 1♂ (MABIK CR00258876), dissected and mounted on 2 H-S slides; and • 1♂ (MinRB-Hr104-L002) and 1♂ (MABIK CR00258873), each preserved in a vial with 95% ethanol. All material was collected from the type locality.

#### Other material examined.

• 1♀ (MInRB-Hr104-S004), dissected and mounted on 2 H-S slides, collected from washings of marine plastic debris (expanded polystyrene buoy) at Chagwido Port, Jeju (33°19'25.86"N, 126°09'56.88"E) on 20 September 2022 by GH Han.

#### Description.

Female. Body length 441–535 μm (*n* = 6; measured from anterior margin of rostrum to posterior margin of caudal rami). Habitus broad, moderately flattened and gradually tapering posteriorly in dorsal view, separation between prosome and urosome indistinct (Fig. 1A). Cephalothorax bell-shaped, slightly broadening posteriorly, maximum width 197 µm at posterior margin; dorsally with several paired sensilla, diminutive spinules, and reticulation (observable under high magnification); posteriorly margin fringed with small spinules. Cephalothorax and pedigerous somites laterally ornamented with minute spinules. Free pedigerous somites with dense spinules and scattered sensilla along posterior margins; also, with particular reticulation (observable under high magnification). Urosomites (Figs 1A, 3A) posteriorly fringed with minute spinules (except anal somite); dorsal and ventral surfaces ornamented with transverse rows of fine spinules. Genital double-somite with a transverse spinular row dorsally and laterally, indicating original segmentation; ventrally completely fused. Genital field located near anterior margin of genital double-somite; either side of genital field covered by lobe derived from P6, bearing two setae; each lobe with a small triangular process medially. Anal somite subequal in length to caudal rami, with spinular ornamentation; anal operculum bearing rows of diminutive spinules and pair of sensilla.

Caudal rami (Figs 1A, 3A) ~1.25 × as long as wide; each ramus with seven setae and a ventral tube pore: seta I bare and shortest; setae II and III bare, the latter 1.5 × as long as the former; setae IV and V well-developed, basally fused, and bipinnate; seta VI unipinnate, shorter than seta II; and seta VII tri-articulate, inserted in the distal third.

Rostrum (Fig. 1B, C) completely defined basally, triangular, with a pair of sensilla subdistally; midventral tube pore in subapical position; dorsal surface with reticulate ornamentation.

**Figure 2.**
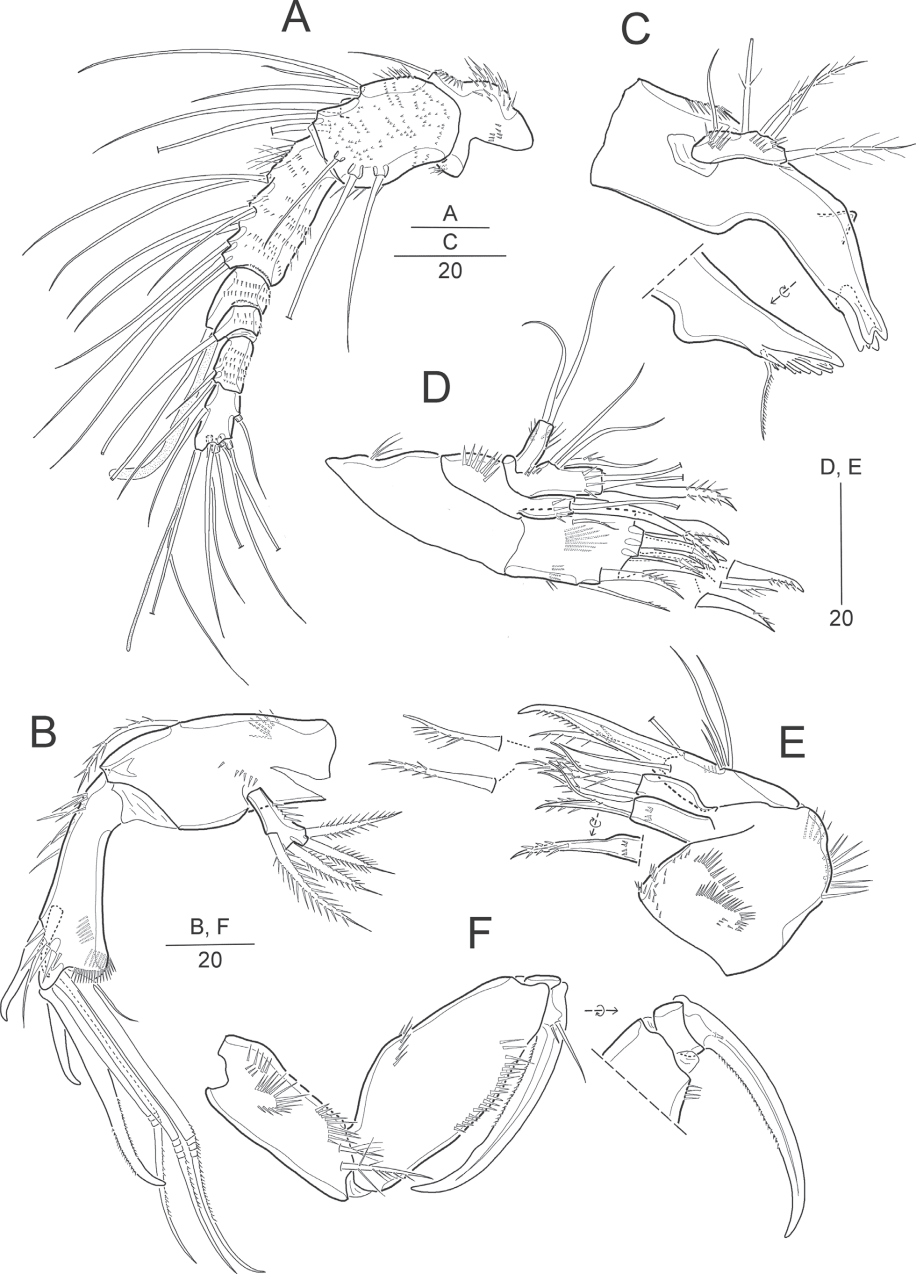
*Pseudonychocamptus setadefectus* sp. nov. Paratype (MABIK CR00258874), female. A. Antennule; B. Antenna; C. Mandible; D. Maxillule; E. Maxilla; F. Maxilliped. Scale bars are in μm.

Antennule (Fig. 2A) 7-segmented; segments 2–6 with several rows of diminutive spinules present on dorsal surface. First segment with several inner spinular rows; second and third segments with inner long spinules. Apical acrothek consisting of an aesthetasc basally fused to two slender setae. Armature formula: 1-[1], 2-[9], 3-[8], 4-[1 + (1 + ae)], 5-[1], 6-[2], 7-[7 + acro].

Antenna (Fig. 2B) 3-segmented. Coxa (not figured) small. Allobasis with one abexopodal seta located at distal third of segment length, and with two rows of spinules. Endopod with a proximal inner tuft of spinules and two subapical frills; laterally with two spines and one slender seta; distally with two strong spines and three geniculate setae (of which most outer one basally fused to a short seta). Exopod 1-segmented, small, ~3 × as long as wide, with four pinnate setae.

Mandible (Fig. 2C) with well-developed gnathobase bearing several blunt teeth around distal margin and one pinnate seta at distal corner. Mandibular palp small, uniramous, with one (exopodal) bare and three (endopodal) pinnate lateral setae, and one (basal) plumose apical seta ~1.7–1.8 × length of palp.

Maxillule (Fig. 2D). Praecoxa with few spinules along outer margin; arthrite with a slender seta on anterior surface and eight elements around distal margin; with one row of long spinules on posterior surface. Coxa and basis each with one cylindrical endite, two spinular rows on anterior surface. Coxal endite with one slender seta and one pinnate spine. Basal endite with two slender setae and one pinnate spine. Endopod incorporated into basis, represented by three setae (one pinnate, two bare). Exopod 1-segmented, cylindrical, with two apical setae and two surface rows of fine spinules.

Maxilla (Fig. 2E). Syncoxa; ornamented with one row of long spinules along distal outer edge, several rows of spinules on posterior surface, and minute spinules around inner margin; with two endites, each with one bipinnate spine (spine on proximal endite fused to segment) and two pinnate setae. Allobasis produced into a stout, distally pinnate claw, accompanied by one pinnate and two bare setae. Endopod incorporated into allobasis, consisting of two long basally fused setae and two short elements.

Maxilliped (Fig. 2F). Syncoxa with two pinnate setae and several rows of spinules. Basis longer than syncoxa, outer margin slightly convex, with one transverse row of spinules; palmar margin bearing a longitudinal row of spinules. Endopod 1-segmented, with a long, curved, proximally pinnate claw; with two anterior accessory setae (one small, one long) and one posterior tube pore.

**Figure 3.**
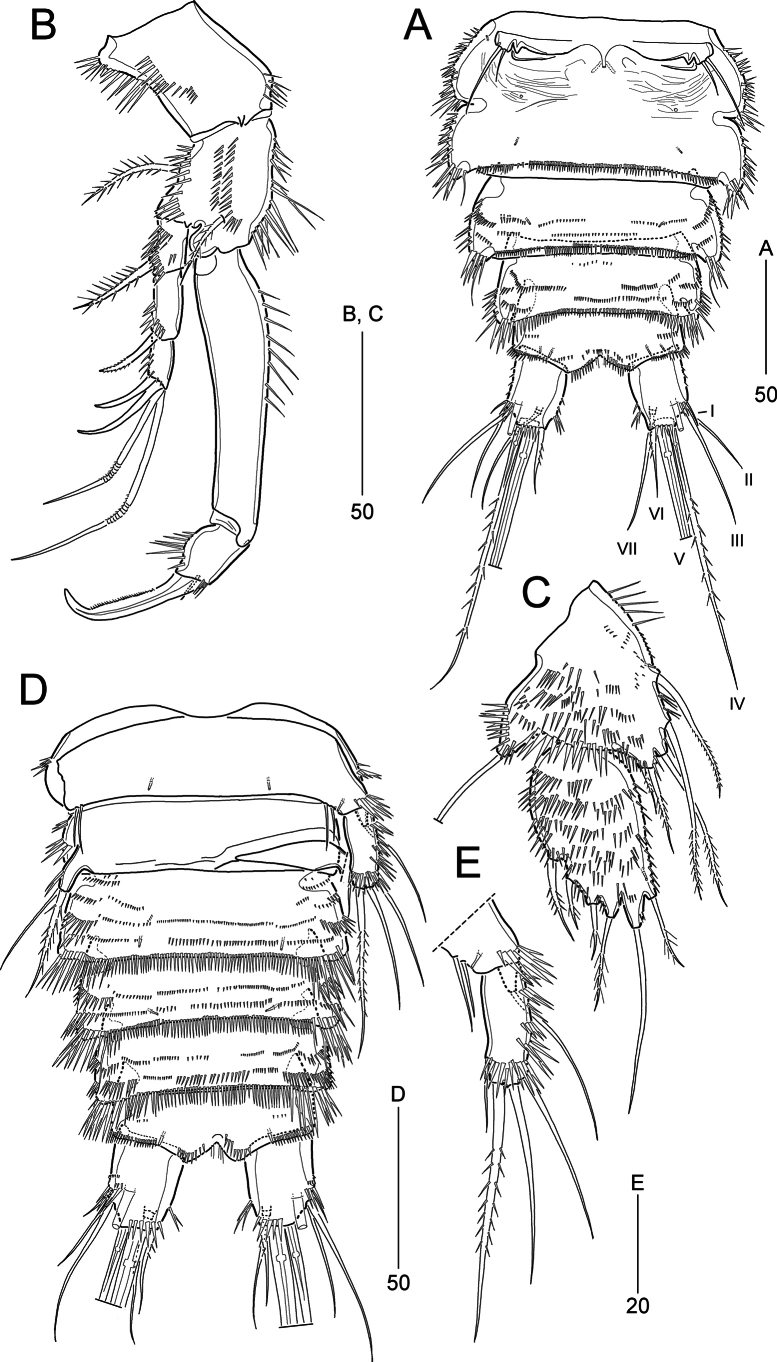
*Pseudonychocamptus setadefectus* sp. nov. Paratype (MABIK CR00258874), female: A. Urosome, ventral; B. P1, anterior; C. P5; Paratype (MABIK CR00258876), male: D. Urosome, ventral. Scale bars are in μm.

**Figure 4.**
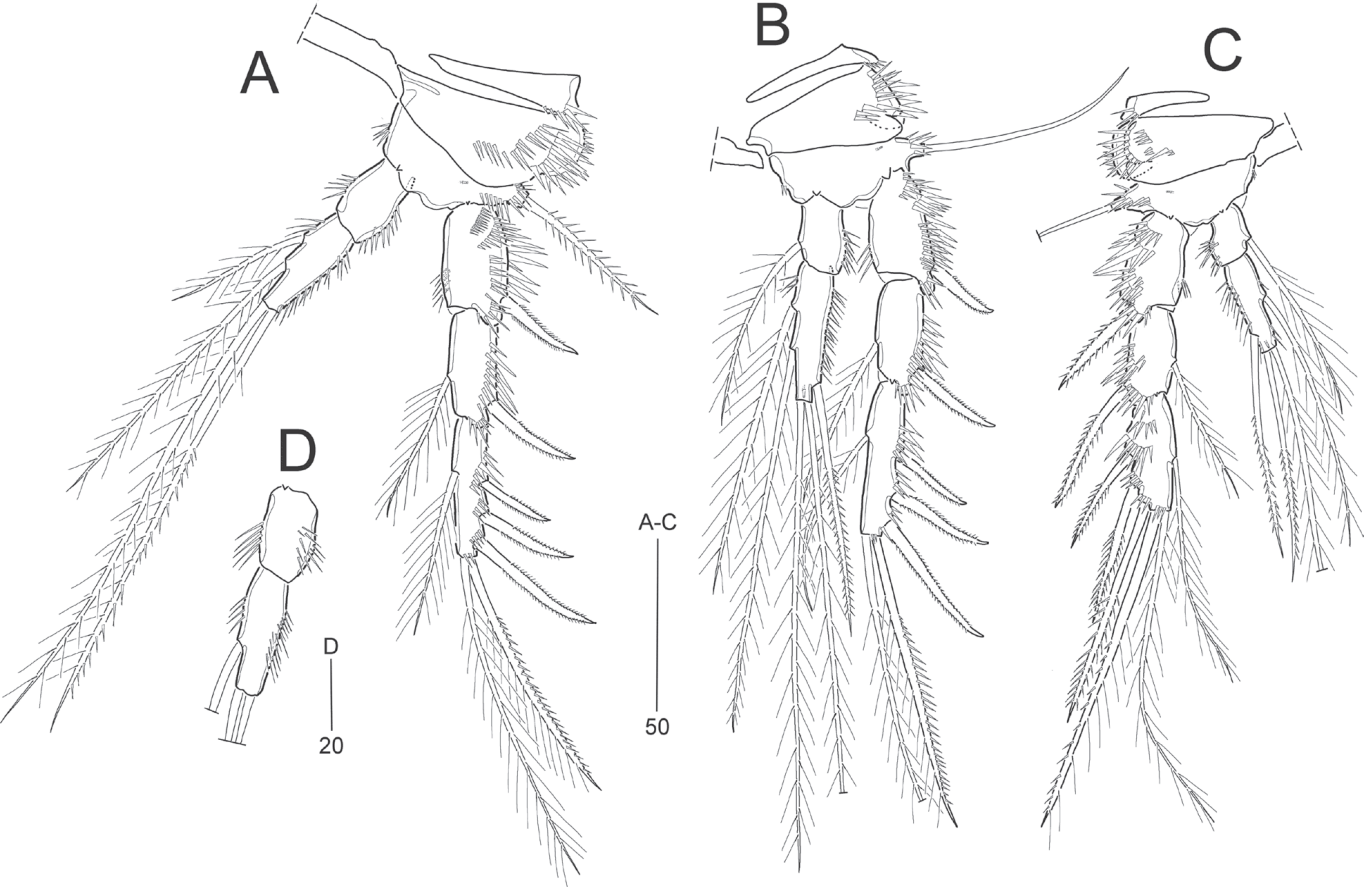
*Pseudonychocamptus setadefectus* sp. nov. Paratype (MABIK CR00258874), female. A. P2, anterior; B. P3, anterior; C. P4, anterior; D. Abnormal of P2 enp-2. Scale bars are in μm.

P1–P4 (Figs 3B, 4A–C) with wide and narrow intercoxal sclerite (not figured for P1); all legs with 3-segmented exopods and 2-segmented endopods.

P1 (Fig. 3B). Coxa large, longer than wide, with several spinular rows on surface. Basis as large as coxa, with one bipinnate seta on outer margin and several spinules, and with one bipinnate seta arising near insertion of endopod. Exopod reaching middle of enp-1, 3-segmented: exp-1 with one bipinnate outer seta; exp-2 with one serrate outer spine; exp-3 with two bare outer spines and two geniculate apical setae. Endopod 2-segmented; enp-1 elongated, ~1.7 × as long as exopod, with long spinules along proximal half of inner margin sparsely; enp-2 short, with one strong, serrate claw and one tiny seta; various-sized spinules along outer margin and few short spinules around inner distal corner.

P2–P4 (Fig. 4A–C). Praecoxae small, transversely elongated-triangular, with several spinules around outer corner. Coxae with several spinular rows along outer margin and on anterior surface. Bases with few spinules on inner margin, several spinules around outer distal corner, and one tube pore on anterior surface. Outer margin of basis with one bipinnate spiniform seta (P2) or one bare seta (P3 and P4). Exopods longer than endopods, gradually tapering distally (especially in P2 and P3); each segment ornamented with spinules along outer and anterior margins; exp-1 with inner spinules subdistally; exp-3 longest. Enp-1 shorter than enp-2, with spinules along outer margin and few (P2) or no spinules (P3 and P4) on inner margin, and with one inner seta in P3 and P4 enp-1 (absent in P2); enp-2 with several spinules along outer margin and few spinules near insertion of proximal seta, and with one (P4) or two (P2 and P3) inner setae, two apical setae and one outer seta in P3 and P4 (absent in P2). P3 and P4 enp-2 with a tube pore near distal margin.

Armature formula of P1–P4 as follows:

**Table:** 

	Exopod	Endopod
P1	0.0.022	0.020
P2	0.1.123	0.1-220 [0.120 in ♂]
P3	0.1.123	1.221 [0.120 in ♂]
P4	0.1.123	1.121 [020 in ♂]

P5 (Fig. 3C). Baseoendopod and exopod separate; anterior surface of both segments covered with spinules (ornamentation of exopod denser than that of baseoendopod). Baseoendopod with one bare basal seta arising from a short and thick setophore; endopodal lobe reaching to proximal fifth of exopod, bearing five bipinnate setae, outermost shortest. Exopod oval-shaped, ~1.8 × as long as wide, bearing one bare and five pinnate setae, of which bare one longest and located terminally.

Male (based on the paratypes). Body (Fig. 1D) smaller than female. Body length 349–400 μm (*n* = 3; measured in the same way as in females), maximum width 148 µm at posterior margin of cephalothorax. Sexual dimorphism present in genital segmentation, antennule, P2–P6.

Urosome (Fig. 3D) narrower than in female, 6-segmented; genital somite and ﬁrst abdominal somite completely separate. Second to fourth abdominal somites with a row of strong spinules along posteroventral margins. Caudal rami as in female.

**Figure 5.**
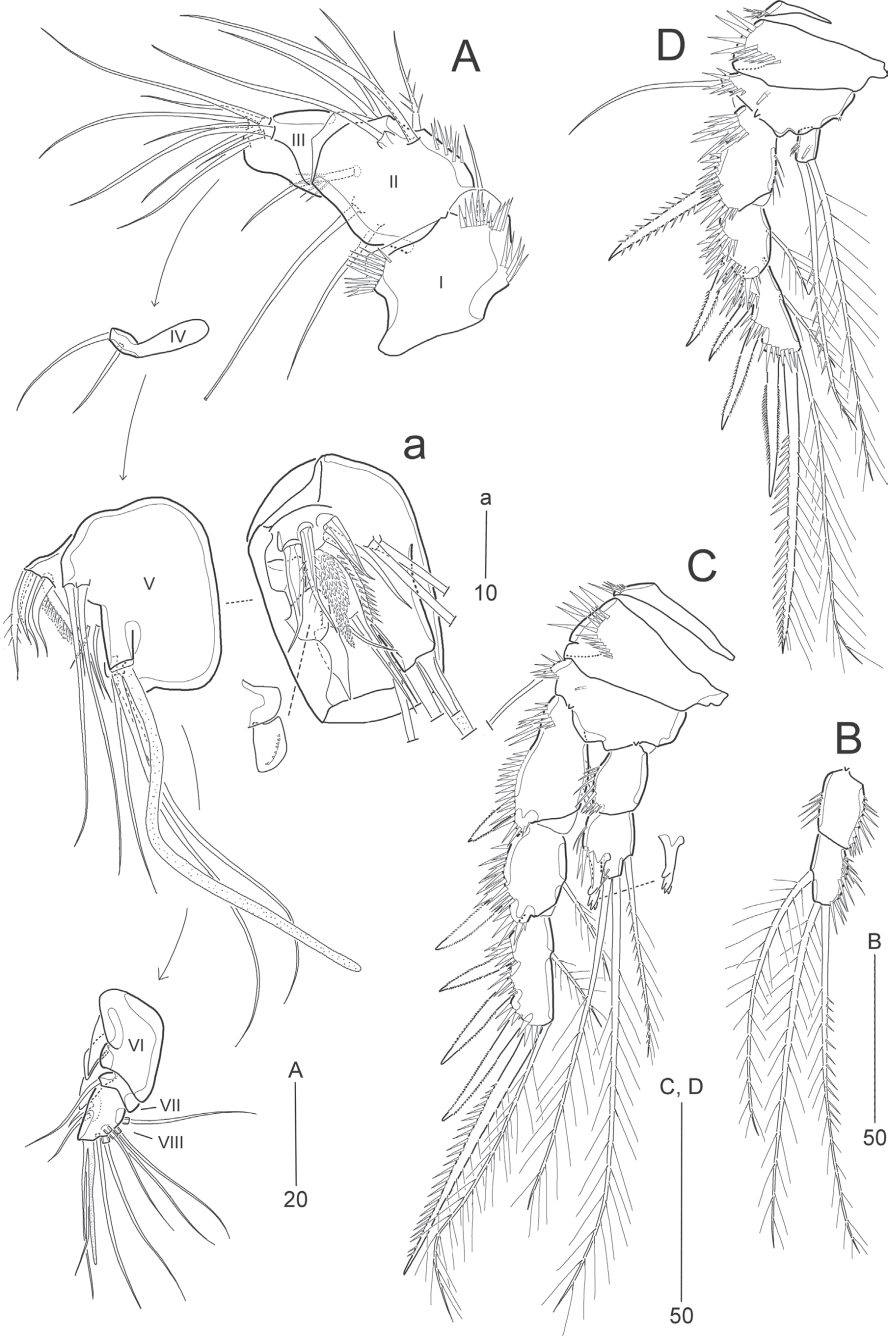
*Pseudonychocamptus setadefectus* sp. nov. Paratype (MABIK CR00258876), male. A. Antennule a Fifth segment of antennule, inner; B. P2 endopod, anterior; C. P3, anterior; D. P4, anterior. Scale bars are in μm.

Antennule (Fig. 5A) 8-segmented, subchirocer, geniculate between segments 5 and 6. First segment as in females; second segment with nine setae, including two pinnate; fifth segment swollen (Fig. 5a); eighth segment triangular. Armature formula: 1-[1], 2-[9], 3-[8], 4-[2], 5-[9 + 2 pinnate + 2 modified + 2 teethlike elements + (1 + ae)], 6-[3 elements], 7-[1], 8-[8 + acro].

Swimming legs. P1 and P2 similar to those of female, except for P2 enp-1 and enp-2 subequal in length (enp-2 relatively shorter than that of female), with one inner and two apical setae (Fig. 5B).

P3 and P4 (Fig. 5C, D). Exopods more robust than those of females. P3 exp-3 considerably shorter, with thicker spines. P3 endopod not reaching to distal margin of exp-2; enp-1 without inner seta; enp-2 with one inner and two apical setae and one anterior tube pore; outer lateral margin produced into a short apophysis bearing distal cuspidate tip; coarse spinules present near at base of apophysis. P4 endopod reduced, 1-segmented, with two apical setae and one anterior tube pore.

P5 (Fig. 3D, E). Left and right baseoendopods fused medially, each with one outer basal seta; endopodal lobe vestigial, represented by two bare setae; posterior margin with two tube pores. Exopod 1-segmented, oblong, with one inner, one apical and two outer setae; inner seta shortest; spinules present on outer margin and anterior surface.

P6 (Fig. 3D) asymmetrical, one side functional, the other fused to somite; outer sides of both plates produced into cylindrical processes, each bearing one inner pinnate and one outer bare seta.

#### Variability.

Morphological variation was observed in the armature formula of the thoracopods in *P. setadefectus* sp. nov. In females, the P2 enp-2 typically bears two inner setae; however, in one of the five specimens examined, three inner setae were present.

#### Etymology.

The species name *setadefectus* is derived from the Latin words *seta* (bristle) and *defectus* (lacking, missing), referring to the absence of the proximal endite bearing a seta of maxilla—a distinguishing feature of this species among its congeners. It is an adjective in the singular nominative, gender masculine.

#### Remarks.

Species of the genus *Pseudonychocamptus* exhibit marked sexual dimorphism, particularly in the following: the inner seta on P3–P4 enp-1, present in females, is absent in males; and the two-segmented P4 endopod in females is reduced to a single segment bearing two setae in males. Notably, the latter trait represents the most prominent apomorphy of the genus (Huys and Lee 2009). The newly discovered species from Korea exhibits both of these sexually dimorphic characteristics, thereby supporting its assignment to this genus.

The most distinctive feature of *P. setadefectus* sp. nov., which sets it apart from its six congeners, is the presence of only two endites on the maxilla, lacking the proximal endite represented by a single seta that is present in all other species of the genus. This condition represents a unique and highly unusual trait within *Pseudonychocamptus*.

The new species, *P. setadefectus* sp. nov., shares the presence of two inner setae on the female P3 enp-2 with four of the six valid species in the genus. These species are *P. abbreviatus* (Sars G.O., 1920), *P. colomboi* Ceccherelli, 1988, *P. marinovi* Apostolov & Petkovski, 1980, and *P. proximus* (Sars G.O., 1908). Conversely, *P. koreni* (Boeck, 1873) and *P. spinifer* Lang, 1965 possess only one inner seta on the female P3 enp-2. Among these, *P. setadefectus* sp. nov. is most similar to *P. colomboi* in that the male P2–P3 enp-2 bears only a single seta. It should be noted, however, that the male of *P. marinovi* has not yet been described. Previous studies (e.g., Ceccherelli 1988; Huys and Lee 2009) indicate that *P. marinovi* can be distinguished from the other three species (*P. abbreviatus*, *P. colomboi*, and *P. proximus*) by the broader than long caudal rami.

Although the morphological differences between females of *P. setadefectus* sp. nov. and *P. colomboi* are relatively subtle, the new Korean species can be readily distinguished by several characters. The fourth outermost seta on the P5 exopod is ~1.5 × longer than the third outermost seta in *P. setadefectus* sp. nov. (Fig. 3C), whereas these setae are subequal in length in *P. colomboi* (Ceccherelli 1988: fig. 8F). Additionally, the apical (basal) seta on the mandibular palp in *P. setadefectus* sp. nov. is 1.7–1.8 × as long as the total length of the palp (Fig. 3D), compared to *P. colomboi*, in which it exceeds twice the palp length (Ceccherelli 1988: fig. 7H). In males, the P5 exopod of *P. setadefectus* sp. nov. possesses an inner seta (Fig. 3D), which is absent in *P. colomboi* (Ceccherelli 1988: fig. 9G). Furthermore, the P6 is asymmetrical in *P. setadefectus* sp. nov. (Fig. 3D), whereas it is symmetrical in *P. colomboi* (Ceccherelli 1988: fig. 9A).

In addition to the aforementioned differences, several minor features further distinguish *P. setadefectus* sp. nov. from *P. colomboi*: the maxillary endopod bears four setae in *P. setadefectus* sp. nov., but three in *P. colomboi*; the maxillipedal claw is accompanied by two accessory setae in the new species, versus one in *P. colomboi*; the inner setae on the female P2 enp-2 differ in relative length—the distal seta is ~2 × as long as the proximal in *P. setadefectus* sp. nov., whereas their length ratio is ~3:1 in *P. colomboi*; all setae, except for the longest apical seta, on the female P5 exopod are ornamented in *P. setadefectus* sp. nov., but all are bare in *P. colomboi*; the two setae of the female P6 differ in length by more than twofold in *P. setadefectus* sp. nov., but they are subequal in *P. colomboi*; the male P2 enp-1 and enp-2 are subequal in length in *P. setadefectus* sp. nov., whereas the enp-2 is slightly shorter in *P. colomboi*; and *P. setadefectus* sp. nov. possesses a single pair of sensilla near the apex of the rostrum, compared to two pairs in *P. colomboi*.

##### ﻿Genus *Heterolaophonte* Lang, 1948

### ﻿ Heterolaophonte discophora

Taxon classificationAnimaliaHarpacticoidaLaophontidae

(Willey, 1929)

D4452754-2E3A-5569-872A-9A0EE2E42F0C

Figs 6, 7, 8, 9

Laophonte discophora Willey, 1929: 531, Abb. 2, 3, 6; Willey 1930: 607, pl. XVIII, figs 16~18 (cited from Itô 1974); Willey 1931: 5.
Heterolaophonte discophora Lang, 1948: 1375, fig. 557-1; Lang 1965: 480, figs 262–265; Itô 1974: 628–638, figs 42–47; Song 2000: 83–86, fig. 25; Kim 2013: 25–27, fig. 9.
Heterolaophonte rotundipes Chappuis, 1958: 420, figs 23–34.

#### Material examined.

(1) • Gohado (34°45'56.04"N, 126°22'22.38"E), Mokpo, Jeollanam-do, marine rope debris; 01 April 2021; leg. OH Yu, SL Kim and SM Kang: 1♀ (MinRB-Hr105-L001), 3♀♀ (MABIK CR00258880), 1♂ (MinRB-Hr105-L002) and 3♂♂ (MABIK CR00258881), preserved separately in a vial with 95% ethanol; 1♀ (MinRB-Hr105-S003), 2♀♀ (MABIK CR00258882–00258883), 1♂ (MInRB-Hr105-S006) and 2♂♂ (MABIK CR00258884–00258885), each dissected and mounted on 1 or 2 H-S slides.

(2) • Naechi Beach (34°53'17.94"N, 126°00'05.68"E), Sinan, Jeollanam-do, expanded polystyrene buoy; 14 September 2022; leg. GH Han: 1♀ (MABIK CR00258877), dissected and mounted on 2 H-S slides. • Same locality, fishing net; 01 February 2024; leg. SL Kim and SJ Gwak: 5♀♀ (MinRB-Hr105-L003), preserved in a vial with 95% ethanol.

(3) • Masan-ri (36°00'58.99"N, 129°29'05.55"E), Pohang, Gyeongsangbuk-do, green algae; 27 February 1997; leg. J Lee; 1♂ (MinRB-Hr105-L004), preserved in a vial with 95% ethanol. • Same area, coralline algae (36°00'59.90"N, 129°28'58.32"E); 23 April 2005; leg. J Lee: 1♀ (MABIK CR00258878, asymmetry in the number of setae on P2 enp-2), dissected and mounted on 2 H-S slides and 1♀ (MABIK CR00258879), preserved in a vial with 95% ethanol. • Same area (36°00'59.90"N, 129°28'58.32"E), coralline algae; 26 April 2005; leg. J Lee: 2♀♀ (MinRB-Hr105-L005, asymmetry in the number of setae on P2 enp-2), preserved in a vial with 95% ethanol.

#### Redescription.

**Figure 6.**
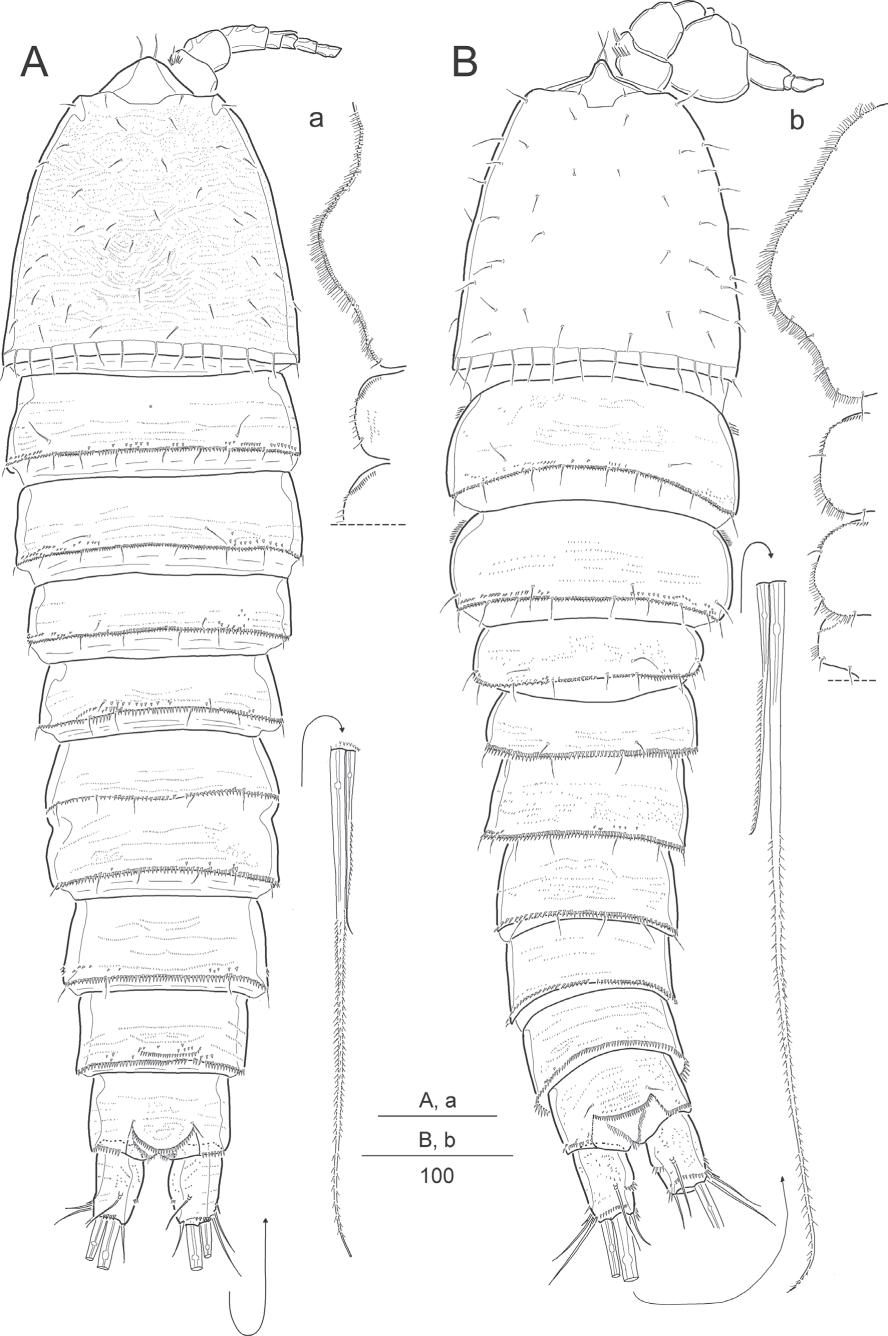
*Heterolaophonte discophora* (Willey, 1929), female: A. Habitus, dorsal a lateral margin of cephalothorax and P2–P3-bearing somites; male: B. Habitus, dorsal b lateral margin of cephalothorax and P2–P4-bearing somites. Scale bars are in μm.

**Figure 7.**
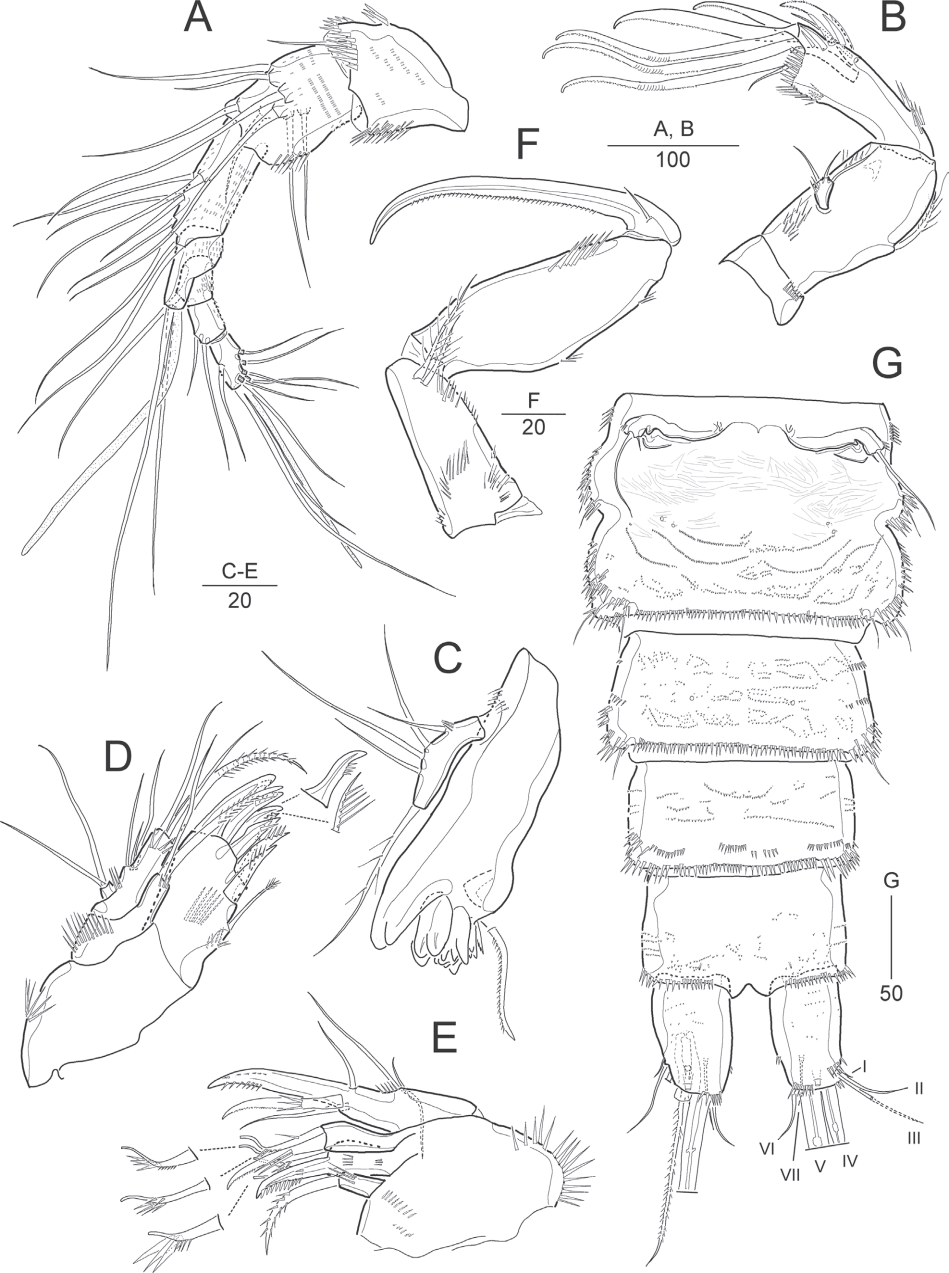
*Heterolaophonte discophora* (Willey, 1929), female. A. Antennule; B. Antenna; C. Mandible; D. Maxillule; E. Maxilla; F. Maxilliped; G. Urosome, ventral. Scale bars are in μm.

Female. Body length 827–1,098 μm (*n* = 17; measured from anterior margin of rostrum to posterior margin of caudal rami). Habitus slender, cylindrical, and gradually tapering posteriorly in dorsal view, separation between prosome and urosome indistinct (Fig. 6A). Cephalothorax with diminutive spinules and several sensilla dorsally; posterior margin smooth, bearing several sensilla. Free pedigerous somites with diminutive spinular rows on dorsal surface, and one or two rows of spinules and several sensilla along posterior margin. Ventrolateral margins of cephalothorax fringed with a closely set of spinules (Fig. 6a). Urosomites (Figs 6A, 7G) with spinules along posterior margin (except anal somite); dorsal and ventral surfaces with patterns of diminutive spinules. Genital double-somite with a transverse spinular row dorsally and laterally, indicating original segmentation; ventrally completely fused. Genital field located near anterior margin of genital double-somite; each side covered by lobe derived from P6, bearing one inner vestigial and one outer long seta; small triangular process present medially on each lobe. Anal somite as long as caudal rami, ornamented with tiny dorsal spinules and lateral/ventral spinules along posterior margin; semicircular operculum ornamented with marginal spinules and pair of sensilla.

Caudal rami (Figs 6A, 7G) ~1.7 × longer than wide; with seven setae: seta I bare, diminutive, identified at high magnification; setae II and III bare, the former shorter than the latter; setae IV and V well-developed, fused basally to each other, and bipinnate; seta VI bare, shorter than seta II, seta VII tri-articulate, arising dorsally in distal 1/3 of ramus.

Rostrum (Fig. 6A) completely defined basally, triangular, with pair of sensilla near apex; midventral tube pore in subapical position (not figured).

Antennule (Fig. 7A) 7-segmented; segments 1–5 covered with minute spinules on dorsal view. First segment short, with several spinular rows; second segment with outer distal spinules; third segment longest; fourth segment with one seta fused basally to an aesthetasc on a distal peduncle; seventh segment tapering distally, with apical acrothek composed of one aesthetasc and two long setae. Armature formula: 1-[1], 2-[8], 3-[7], 4-[1 + (1 + ae)], 5-[1], 6-[2], 7-[7 + acro].

Antenna (Fig. 7B) 3-segmented, comprising coxa, allobasis, and 1-segmented endopod. Coxa small, with a row of spinules. Allobasis with 1 abexopodal seta located approximately midway along outer margin of segment, and with long setules near base of exopod. Endopod as long as allobasis, ornamented with long spinules along inner proximal margin and rows of spinules along outer distal margin; lateral armature consisting of two pinnate spines and one slender seta; distal armature composed of two strong spines and three geniculate setae (of which most outer one basally fused to short seta). Exopod small, 1-segmented, with three slender setae, middle one shortest inner one longest.

Mandible (Fig. 7C). Coxa transversely elongated; gnathobase with one bicuspidate, three multicuspidate teeth, and one pinnate spine around distal margin and one pinnate seta at distal corner. Palp small, uniramous, with four bare (1 exopodal, 3 endopodal) lateral setae and one pinnate apical (basal) seta; with few small spinules present at base of proximal lateral seta.

Maxillule (Fig. 7D). Praecoxa with a row of outer spinules; arthrite with one subdistal and eight distal elements; posterior surface with a row of spinules. Coxa with a row of long spinules on anterior surface and few spinules around inner distal margin; cylindrical endite bearing one spinulose and one slender seta. Basis with one anterior spinular row; endite with one spinulose and two slender setae. Endopod nearly incorporated into basis, represented by three bare setae, of which middle one smaller than others. Exopod small, 1-segmented, with two long apical setae.

Maxilla (Fig. 7E). Syncoxa with a long spinular row around outer margin and two rows of small spinules on surface; with three endites: proximal endite (praecoxal) bearing one pinnate seta; middle endite with one serrate spine and two pinnate setae, of which spine fused basally to segment; distal endite with one spine and two pinnate setae. Allobasis drawn out into a stout claw, with one spine (bearing three spinules near midlength) and two slender setae. Endopod represented by three bare setae (1 short, 2 long).

Maxilliped (Fig. 7F) 3-segmented, comprising syncoxa, basis, and 1-segmented endopod. Syncoxa with two distal plumose setae and several spinular rows. Basis longer than preceding segment, outer margin slightly convex, with two rows of spinules; inner margin with a row of strong spinules in distal half. Endopod 1-segmented, with a long, curved claw; with one short bare seta anteriorly and a tube pore posteriorly (as in *P. setadefectus* sp. nov., Fig. 2F); concave inner margin bearing row of spinules.

**Figure 8.**
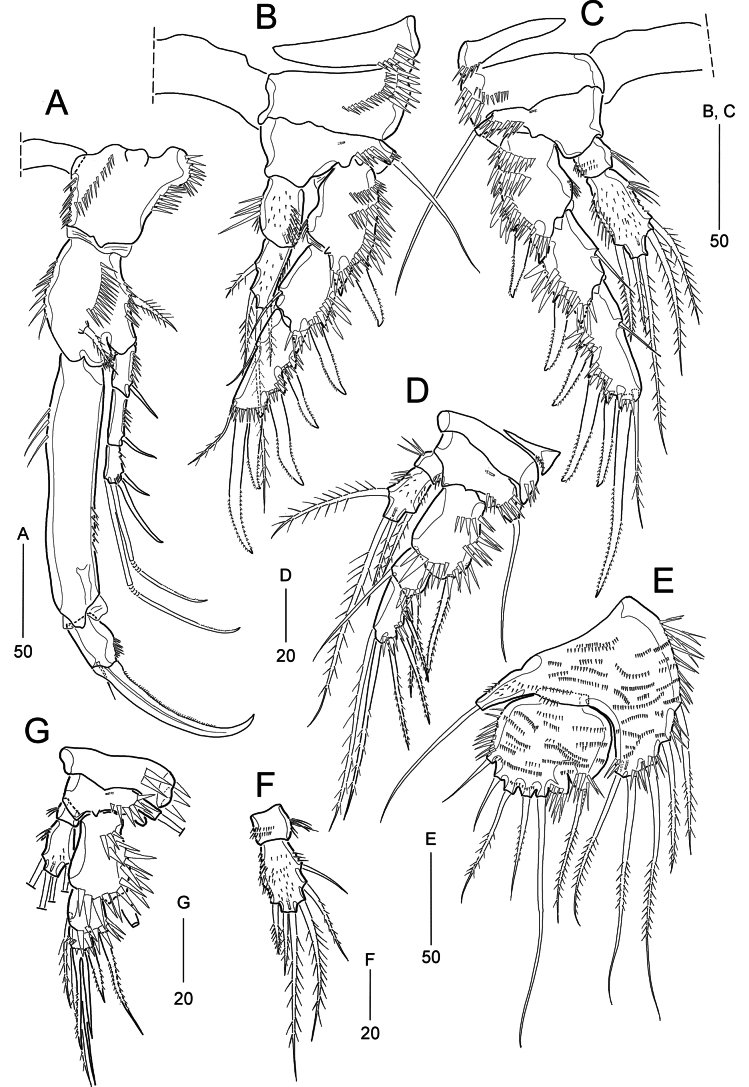
*Heterolaophonte discophora* (Willey, 1929), female. A. P1, anterior; B. P2, anterior; C. P3, anterior; D. P4, anterior; E. P5; F. P3 endopod from Masan-ri specimen, anterior; G. Abnormal of P4 from Naechi specimen, anterior. Scale bars are in μm.

P1 (Fig. 8A). Intercoxal sclerite transversely elongated and narrow. Praecoxa damaged (not shown). Coxa large, proximal outer margin protruded, with spinular rows along inner and outer margins. Basis with three spinular rows; one outer and one inner seta (displaced anteriorly). Exopod shorter than enp-1, 3-segmented, each segment with outer spinules; exp-1 and exp-2 with one outer spine; exp-2 longest; exp-3 with two outer spines and two geniculate apical setae. Endopod 2-segmented, enp-1 elongated, ~2 × as long as entire exopod; enp-2 short, bearing one strong, serrate claw and one small seta, and several spinules along outer margin.

P2 and P3 (Fig. 8B, C). Intercoxal sclerites subrectangular. Praecoxae transversely elongated triangular, with outer spinular rows. Coxae and bases ornamented with spinular rows along outer margin and on anterior surface. Bases each with one bare outer seta and one anterior tube pore. Exopods 3-segmented, longer than endopods; each segment ornamented with strong spinules as illustrated; exp-2 shortest, exp-3 longest; exp-1 with one outer spine; exp-2 with one outer spine and one inner seta; exp-3 with three outer spines, one apical spine, one apical seta, and one inner seta. Endopods 2-segmented, enp-1 as long as enp-2 in P2, but much shorter than enp-2 in P3, with long inner spinules and short outer spinules, with (P2) or without (P3) one tube pore on outer corner; enp-2 with spinules along outer margin and on anterior surface (P3); with one tube pore located proximally (P2) or distally (P3), bearing two inner (occasionally 3 in P3), two apical and no outer (P2) or one (P3) outer seta.

**Figure 9.**
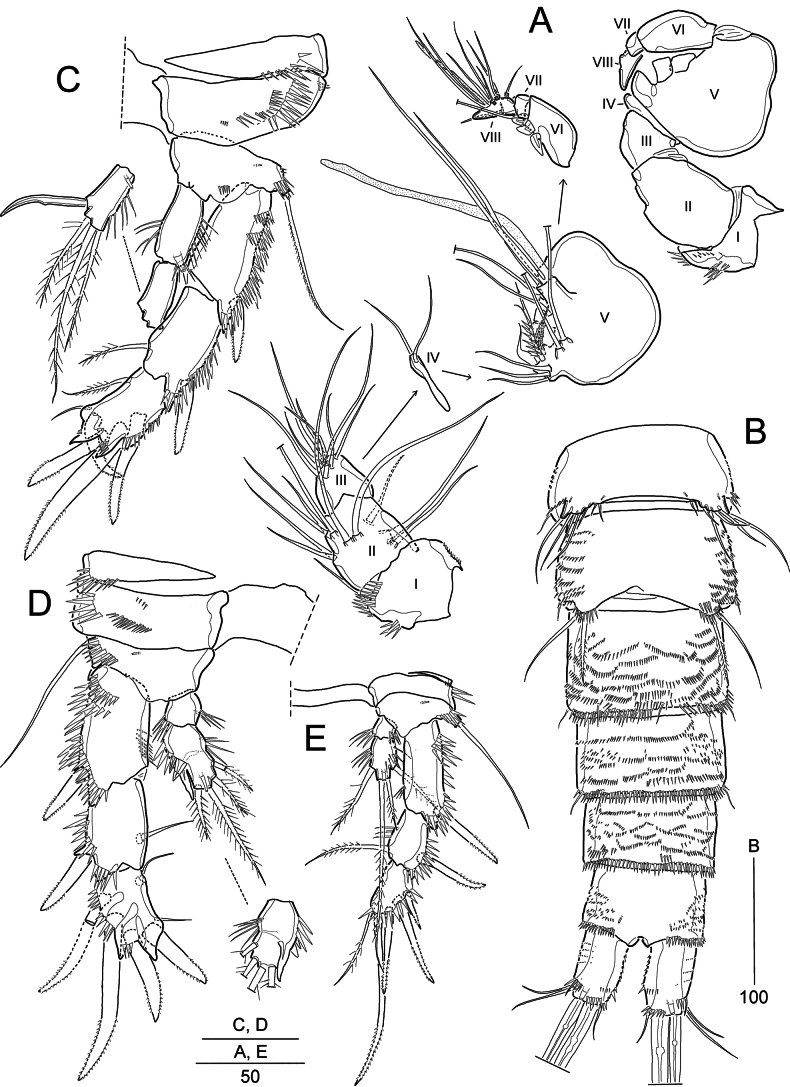
*Heterolaophonte discophora* (Willey, 1929), male. A. Antennule; B. Urosome, ventral; C. P2, anterior; D. P3, anterior and P3 enp-2 ﬁgured separately, posterior; E. P4 anterior. Scale bars are in μm.

P4 (Fig. 8D). Intercoxal sclerite elongated and narrow (see Fig. 9E). Praecoxa small, transversely elongated and triangular. Coxa and basis with spinular rows along outer margins and on anterior surface. Basis with one bare outer seta and one anterior tube pore. Exopod 3-segmented, gradually tapering distally, longer than endopod; exp-1 broad, with several spinular rows on anterior surface; exp-2 shortest, with one outer, slender, and elongated spine and one inner seta; exp-3 with three outer, slender, and elongated spines, and two apical elements. Endopod 2-segmented, enp-1 with long inner spinules; enp-2 ~2.5 × as long as enp-1, with spinules on anterior surface, bearing one inner, two apical, and one outer seta.

Armature formula of P1–P4 as follows:

**Table:** 

	Exopod	Endopod
P1	0.0.022	0.020
P2	0.1.123	0.220
P3	0.1.123	0.2–321[0.220 in ♂]
P4	0.1.023	0.121

P5 (Fig. 8E) with baseoendopod and 1-segmented exopod, both surfaces covered with fine spinules. Baseoendopod with one bare basal seta arising from long cylindrical setophore; endopodal lobe extending to distal third of exopod, bearing spinules along inner margin, and armed with three inner and two distal bipinnate setae. Exopod widely subquadrate with slightly convex inner margin, strongly sclerotized and dented between innermost distal seta and longest seta; with three bare and three pinnate setae.

Male. Body (Fig. 6B) generally similar to that of female, but with a more or less slender urosome. Body length 688–927 μm (*n* = 15; measured in the same way as in females). Cephalothorax and pedigerous somites with ornamentation patterns as in females (Fig. 6B, b). Sexual dimorphism in genital segmentation, antennule, P2–P6.

Urosome (Figs 6B, 9B) 6-segmented; genital somite and ﬁrst abdominal somite completely separate; ornamentation as figured.

Antennule (Fig. 9A) 8-segmented, subchirocer, geniculate between segments 5 and 6. First segment with several spinular rows; fourth segment very small; fifth segment swollen; eighth segment triangular. Armature formula: 1-[1], 2-[9], 3-[8], 4-[2], 5-[8+ 1 pinnate + 2 modified + 2 teethlike elements + (1 + ae)], 6-[3 elements], 7-[1], 8-[8 + acro].

P2–P3 (Fig. 9C, D). Exopods broader than in females; exp-3 considerably shorter, with thicker spines. P2 endopod: enp-1 slightly longer than enp-2, with long spinules along inner and outer margin, and one large pore near distal corner; enp-2 with long spinules along outer margin, one tube pore on proximal anterior surface, and inner element modified as strong bare spine. P3 endopod: enp-1 as in females; enp-2 ornamented with long inner spinules and stout spinules along outer margin proximally; bearing one short apophysis on outer lateral margin, two inner and two distal setae (shorter than in females) and one tube pore on distal anterior surface; apophysis weakly curved outwardly at distal tip, bearing one stout and one small strengthen spinules (protuberance ?) near its base.

P4 (Fig. 9E). Exopod considerably longer than in females, with thicker spines. Endopod as in females, except enp-2 bearing tube pore on distal anterior surface.

P5 (Fig. 9B). Left and right baseoendopods medially fused, baseoendopod and exopod fused, forming small lobe bearing seven setae, of which two inner ones diminutive; posterior margin with four tube pores.

P6 (Fig. 9B) symmetrical, represented by small lobe bearing one median spine and one outer seta, and ornamented with spinular row along distal margin.

#### Variability.

Previous descriptions of *H. discophora* (e.g., Lang 1965: fig. 263i; Itô 1974: fig. 44.3) report three inner setae on the female P3 enp-2. However, examination of 17 Korean specimens revealed notable variation in thoracopod setal armature. Thirteen specimens from Gohado and Naechi exhibited only two inner setae on both left and right rami (Fig. 8C). In contrast, left–right asymmetry in the number of inner setae was found exclusively in specimens from Masan-ri, where three of the four individuals examined showed this trait (Fig. 8F), indicating significant intraspecific variability within the species.

#### Abnormality.

In a female specimen from Gohado, caudal seta IV on the right caudal ramus was underdeveloped and deeply embedded within the ramus (Fig. 7G). Additionally, another female specimen exhibited an abnormal P4 exopod. Compared to the normal morphology of P4 (Fig. 8D), the distal segment of the abnormal exopod was markedly shortened and bore five spines/setae, with two distal spines fused together (Fig. 8G).

#### Remarks.

*Heterolaophonte discophora* (Willey, 1929) was originally described by Willey (1929) based on a female specimen collected from St. Andrews, New Brunswick, Canada. The male was subsequently reported from Passamaquoddy Bay, New Brunswick, by Willey (1931). However, these initial descriptions and illustrations were brief and provided limited morphological details, particularly regarding the P5 of both sexes and the P2–P4. Lang (1965) later provide a detailed redescription based on specimens collected from Monterey Bay, near Hopkins Marine Station, and treated *H. rotundipes* Chappuis, 1958, (originally described from Puget Sound near Seattle, Washington, USA) as a junior synonym of *H. discophora*. Following Lang’s taxonomic revision, Itô (1974) also redescribed the species based on male and female specimens from Hokkaido, Japan. Given the incompleteness of the original description, the present comparison of the Korean material relies primarily on the comprehensive accounts by Lang (1965) and Itô (1974).

*Heterolaophonte discophora* was first reported from Korea in the doctoral dissertation by Song (2000). In that study, Song identified specimens collected from macroalgae at Masan-ri, Pohang, as *H. discophora*, providing a brief species description, with the setal formula of P2–P4 and illustrations of the female P1, P4, P5, the A2 exopod, the caudal ramus, and the male P5. He noticed that the Korean *H. discophora* machted Itô’s (1974) redescription in all characters except for the relative lengths of the two short inner setae on the male P5. We obtained Korean specimens of *H. discophora* from MPD stranded along the coasts of Gohado, Mokpo, and Naechi Beach, Sinan, as well as Masan-ri. Contrary to Song’s (2000) observations, our examination of these newly collected Korean specimens reveals additional morphological differences not previously reported.

One of the diagnostic traits of *H. discophora* is the shape of the P4 exp-3, which is slender and elongated, gradually broadening distally in its proximal part (Willey 1929: fig. 2; Lang 1965: fig. 264a). However, this characteristic appears less pronounced in Japanese specimens (Itô 1974: fig. 264a, a.1) and in the Korean material examined here (Fig. 8D). Nonetheless, the markedly large and broad P4 exp-1 in *H. discophora*, relative to exp-2 and exp-3, may represent a more reliable diagnostic feature of this species than the morphology of exp-3 when compared to congeners.

Comparative analysis of the Korean specimens with previous records of *H. discophora* reveals four distinct morphological traits. Lang (1965) reported four setae on the endopodal lobe of the female P5, whereas Chappuis (1958) and Itô (1974) described five; the Korean specimens also possess five setae on this lobe. It is possible that Lang (1965) observed a variable condition of the female P5, considering that most *Heterolaophonte* species have five setae on the corresponding segment, except for *H. exigua* (Scott, 1912), *H. norvegica* Drzycimski, 1968, and *H. tupitskyi* Chislenko, 1976. In the male P5, Lang (1965) and Willey (1931) described five elements, whereas Chappuis (1958) and Itô (1974) noticed seven elements, including two additional small inner setulae—features also observed in the Korean material. It appears that these two inner ornamentations were interpreted as armatures by earlier authors. Regarding the female P3 endopod, Itô (1974) reported three inner setae on the enp-2; however, most Korean specimens only have two, with occasional asymmetry (bearing 2 or 3 inner setae) between the left and right rami (see variability above). Lang (1965) described the male P3 endopod as three-segmented, and Itô (1974) referred to a tripartite condition, though with indistinct boundaries. Conversely, the Korean specimens consistently exhibit a two-segmented endopod, although a posterior suture line is variably developed—absent in some individuals, faint in others, and distinctly visible in a few—potentially leading to interpretative ambiguity. Similar variable chaetotaxy has occasionally been reported in laophontid copepods, particularly in the genus *Quinquelaophonte*, which is closely related to *Heterolaophonte* (Willen 1996; Kim and Lee 2023). Finally, Itô (1974) noted a single triangular, spur-shaped protuberance on the outer distal corner of the male P3 enp-2, which, unfortunately, was not mentioned by Lang (1965). In contrast, the Korean specimens exhibit two triangular, strengthened spinules (protuberances?) of unequal size in the same position, with a similar row of strong spinules also present proximally along the outer margin. This ornamentation was probably overlooked by earlier authors.

In addition, the Korean specimens exhibit several minor differences from previous accounts. They have three setae on the endopod of the maxilla, whereas Lang (1965) described two, and Itô (1974) reported only one. In the female P5 exopod of the Korean material, only three of the five setae are bare, whereas all were described as bare in previous accounts. In the male P4 exopod, the terminal segment is similar in length to the second segment, but it is slightly longer in Lang’s (1965) and Itô’s (1974) material. In the male P2 enp-2, the apical outer seta is longer than the inner distal seta, whereas the reverse condition was described previously. Except for the first difference, these minor disparities are considered herein as intraspecific variability.

## ﻿Discussion

### ﻿Sexual dimorphism in the genus *Pseudonychocamptus*

Sexual dimorphism in male appendages, particularly the swimming legs, has long been recognized as a crucial source of characters for taxonomic identification and phylogenetic inference within the Laophontidae (Lee and Huys 1999; Huys and Lee 2009). Within this context, the genus *Pseudonychocamptus* serves as a clear example, exhibiting consistent and distinctive patterns of sexual dimorphism that aid in clarifying its phylogenetic placement.

The genus *Pseudonychocamptus* comprises a morphologically consistent group of species that aligns well with the current general diagnosis. As noted by Huys and Lee (2009), a prominent apomorphy of the genus is the marked sexual dimorphism of the P4 endopod: females possess a two-segmented endopod with a setal formula of 1,121, whereas in males, it is modified into a small, single segment bearing only two setae. This condition is consistently observed in all known species of the genus, including *P. setadefectus* sp. nov. described herein (except for *P. marinovi*, for which the male remains unknown).

Lee and Huys (1999) proposed a monophyletic clade, named *PWPH*, comprising *Pseudonychocamptus*, *Weddellaophonte* Willen, 1996, *Pilifera* Noodt, 1952, and *Heteronychocamptus* Lee & Huys, 1999, based on shared sexual dimorphism in the P3–P4 enp-1. The importance of male sexual dimorphism in clarifying phylogenetic relationships within the Laophontidae was further emphasized by the discovery of the male of *P. carthyi* Hamond, 1968 (Huys and Lee 2009). Originally placed in *Pseudonychocamptus*, *P. carthyi* was later transferred to the newly established genus *Marbefia* Huys & Lee, 2009, due to its distinctly different P4 endopod morphology in the male. This reassignment highlighted the crucial role of male morphology in defining generic boundaries within the family. In this context, the discovery of the previously unknown male of *P. marinovi* would be indispensable for clarifying the phylogenetic placement of the genus *Pseudonychocamptus*.

### ﻿New insights into morphological characters of *Heterolaophonte* species, with a proposal of four species groups

The genus *Heterolaophonte*, originally established by Lang (1948), comprises a large number of species within the family Laophontidae, with 37 valid species currently recognized (Walter and Boxshall 2025). However, the monophyly of the genus remains unresolved. Historically, the genus was divided into species groups, beginning with the seven groups proposed by Lang (1944, 1948) and later expanded by Vervoort (1964) and Drzycimski (1968). Although these groupings are no longer considered taxonomically valid (Huys 2009; Kaymak and Karaytug 2014), they continue to be used in the literature as a basis for distinguishing species (e.g., Apostolov and Pandourski 2000; Bang et al. 2011; Apostolov 2016). Recently, Kaymak and Karaytug (2014) proposed removing four species—*H. rottenburgi* (Scott, 1912), *H. exigua* (Scott, 1912), *H. australis* (Scott, 1912), and *H. insignis* (Scott, 1914)—from the genus, suggesting they be placed as incertae sedis within the family Laophontidae. They also recommended treating three species and one subspecies—*H. phycobates* (Monard, 1935), *H. pygmaea* (Scott, 1893), *H. tupitskyi* Chislenko, 1976, and *H. curvata micarthros* Marcus & Por, 1960—as species inquirendae within the genus. A comprehensive morphological comparison of sexually dimorphic traits, which provide significant clues for defining phylogenetic relationships among *Heterolaophonte* species, is hindered by the lack of information on the males of ten species: *H. australis* (Scott, 1912), *H. bisetosa* Mielke, 1975, *H. exigua* (Scott, 1912), *H. furcata* Noodt, 1958, *H. insignis* (Scott, 1914), *H. oculata* (Gurney, 1927), *H. phycobates* (Monard, 1935), *H. pygmaea* (Scott, 1893), *H. rottenburgi* (Scott, 1912), and *H. tupitskyi* Chislenko, 1976. Based on morphological analysis, we herein attempt to redefine the species groups within *Heterolaophonte* as a step toward resolving the monophyly of the genus.

Sexual dimorphism in the swimming legs of *Heterolaophonte* can be assessed in the 27 species for which both sexes are known. In these species, male-specific modifications are most frequently observed on the P2 and P3 exopods, with occasional elongation of the P4 exopod compared to females. Two key characteristics are also evident in the P3 endopod: it is generally two-segmented, although some reports describe it as three-segmented—likely reflecting misinterpretation of a weak suture between segments (as discussed in the remarks on *H. discophora*); and the outermost seta on the P3 enp-2 is commonly transformed into an apophysis. Based on these patterns, a character matrix summarizing sexual dimorphism in the swimming legs across the 27 species is presented in Table 1. This matrix serves as the basis for grouping the species into four morphological types according to shared patterns of sexual dimorphism.

**Table 1.** Character matrix summarizing sexual dimorphism in the swimming legs of *Heterolaophonte* species for which both sexes are available (only 27 species). See character definitions below. Abbreviation and symbol: C = character; ? = character state unconfirmed.

	C1	C2	C3	C4	C5	C6	C7	C8	C9	C10	C11
*H. discophora*	1	1	0	1	1	0	0	0	2	1	1
*H. livingstoni*	1	1	0	1	0	0	0	1	2	1	1
*H. tenuispina*	0	1	0	1	0	0	0	1	2	1	1
*H. stroemii*	1	1	0	1	0	0	0	0	2	1	1
*H. murmanica*	1	1	0	?	?	0	0	0	2	1	1
*H. heejinae*	1	1	0	1	1	0	0	0	2	1	1
*H. pauciseta*	1	1	0	1	?	0	0	0	1	1	1
*H. brevipes*	1	1	0	1	1	0	0	0	1	1	1
*H. denticulata*	1	1	0	1	0	0	0	0	1	1	1
*H. serratula*	1	1	0	1	1	0	0	0	1	1	1
*H. norvegica*	1	1	0	?	?	0	0	0	2	1	1
*H. uncinata*	1	1	0	0	0	0	0	0	2	1	2
*H. curvata*	1	1	0	1	1	0	0	0	2	1	2
*H. manifera*	1	1	0	1	?	0	0	0	2	1	2
*H. hamata*	1	1	0	1	1	0	0	1	2	1	2
*H. minuta*	1	1	0	1	0	0	0	0	2	0	0
H. laurentica*	1	1	0	? *	? *	0	0	1	2	0	0
*H. lalanai*	1	1	0	0	1	0	0	2	0	0	0
*H. littoralis*	0	0	1	0	1	1	1	1	2	1	3
*H. longisetigera*	0	0	1	0	1	1	1	1	2	1	3
*H. mendax*	0	0	1	0	1	?	0	1	2	1	3
*H. natator*	0	0	1	0	1	0	1	1	2	1	3
*H. hamondi*	0	0	1	0	1	1	1	0	0**	2	0
*H. letovae*	0	0	1	0	1	1	1	0	0**	2	0
*H. islandica*	0	0	1	0	1	1	1	0	0**	2	0
*H. campbelliensis*	0	1	1	0	1	1	0	1	2	?	?
*H. variabilis*	0	1	1	0	1	1	0	1	2	1	3

* The female specimen of *H. laurentica* described by Nicholls (1941) is likely immature, based on the morphology of P2–P4 (especially P4) and P5. Consequently, comparisons with adult females are not possible, and characters C4 and C5 cannot be reliably assessed. In addition, the outermost element on male P3 enp-2 was described as a spine, rather than an apophysis. ** *H. hamondi*, *H. islandica*, and *H. letovae* lack an inner seta on the female P2 enp-2, and the male P2 enp-2 closely resembles that of the female. Character definitions: C1. Male P2 exopod markedly strengthened: 0 = as in female; 1 = markedly strengthened; C2. Male P3 exopod markedly strengthened: 0 = as in female; 1 = markedly strengthened; C3. Male P4 exopod markedly strengthened: 0 = as in female; 1 = markedly strengthened; C4. Male P4 exopod bearing strongly developed spines: 0 = as in female; 1 = spines strongly developed; C5. Male P4 exopod elongated relative to the female: 0 = as in female; 1 = yes; C6. Male P4 exp-1 extremely elongated: 0 = as in female; 1 = extremely elongated; C7. Male P4 endopod distinctly reduced in size: 0 = as in female; 1 = reduced; C8. Male P3 endopod, three-segmented: 0 = two-segment; 1 = three-segmented; C9. Number of inner setae on female P2 enp-2: 0 = absent; 1 = 1 seta; 2 = 2 setae; C10. Male P2 enp-2 bearing modified inner spine: 0 = absent; 1 = present; C11. Shape of inner spine on male P2 enp-2: 0 = as in female; 1 = elongate; 2 = inflated medially; 3 = short.

These four groups are characterized as follows:

- Group I [15 species: *H. brevipes* Roe, 1958; *H. denticulata* Roe, 1958; *H. discophora*; *H. heejinae* Bang, Lee & Lee, 2011; *H. livingstoni* Apostolov & Pandourski, 1999; *H. murmanica* Letova, 1982; *H. norvegica* Drzycimski, 1968; *H. pauciseta* (Lang, 1936); *H. serratula* Mielke, 1981; *H. stroemii* (Baird, 1837); *H. tenuispina* (Lang, 1934); *H. curvata* (Douwe, 1929); *H. hamata* Jakobi, 1954; *H. manifera* (Wilson C.B., 1932); and *H. uncinata* (Czerniavski, 1868)]: Male P2–P3 exopods are markedly strengthened (Note: Lang (1934) described the male P2 exopod of *H. tenuispina* as identical to that of the female). The male P4 exopod bears strongly developed spines that are distinctly longer or more robust than in the female, although the exopod itself is not markedly strengthened as in P2 or P3. The shape of the inner spine on male P2 enp-2 varies among species: In the first 11 species, it is elongated and distally curved. In the remaining four species (*H. curvata*, *H. hamata*, *H. manifera*, *H. uncinata*), it is distinctly inflated medially.
- Group II [*H. lalanai* Varela & Ortiz, 2008; *H. laurentica* (Nicholls, 1941); *H. minuta* (Boeck, 1873)]: Male P2–P3 exopods are markedly strengthened. The inner seta on the male P2 enp-2 is unmodified and resembles that of the female.
- Group III [*H. campbelliensis* (Lang, 1934) and *H. variabilis* Lang, 1965]: No marked strengthening is observed on the male P2 exopod. The male P3–P4 exopods are markedly strengthened. The P4 exp-1 is conspicuously elongated. The P4 endopod remains similar to that of the female. The presence of a modified inner spine on P2 enp-2 is unconfirmed in *H. campbelliensis*.
- Group IV [*H. hamondi* Hicks, 1975; *H. islandica* Apostolov, 2016; *H. letovae* Huys, 1990; *H. littoralis* (Scott T. & Scott A., 1893); *H. longisetigera* Klie, 1950; *H. mendax* (Klie, 1939); and *H. natator* Lewis, 2022]: No marked strengthening is observed on the male P2 and P3 exopods. The male P4 exopods are markedly strengthened, with exp-1 conspicuously elongated relative to exp-2 and exp-3. The P4 endopod is markedly reduced. A modified inner spine is present on P2 enp-2, except in *H. hamondi*, *H. islandica*, and *H. letovae*, which lack inner setae on the female P2 enp-2 (Table 1).

This morphological classification provides a framework to critically reassess previously proposed autapomorphic features of the genus *Heterolaophonte*, particularly those outlined by Kaymak and Karaytug (2014). These authors suggested three male-specific morphological features as potential autapomorphies: (1) a modified inner spine on the male P2 enp-2, (2) a two-segmented male P3 endopod, and (3) transformation of the inner seta on male P3 enp-2 into a spine. Our comparative analysis reveals that the first character proposed by Kaymak and Karaytug (2014)—a modified inner spine on the male P2 enp-2—is not consistently present across all *Heterolaophonte* species, as it is absent in the three species comprising Group II. Therefore, this feature cannot be regarded as a reliable autapomorphy of the genus. Moreover, comparable but not identical endopodal transformations occur elsewhere in Laophontidae. In several species of *Paralaophonte* Lang, 1948, the distal inner seta of the male P2 enp-2 is transformed into a stout spiniform process (e.g., Wells et al. 1982: fig. 15d; Gómez and Morales-Serna 2013: fig. 36B), whereas in *Galapalaophonte* Fiers, 1991 the apical seta of the male P2 enp-1 is likewise conspicuously modified (e.g., Fiers 1991: figs 15e, 20g, 22h). The second and third features—a two-segmented P3 endopod and the modification of a seta on the male P3 enp-2—are likewise not unique to *Heterolaophonte*. Similar modifications are observed in other laophontid genera (e.g., *Marbefia*, *Quinquelaophonte*, *Pseudonychocamptus*) (Lang 1965; Huys and Lee 2009; Kim et al. 2020; Kim and Lee 2023). Additionally, the third character appears to have been mischaracterized: it is the outer seta, not the inner, that is modified into an apophysis.

These findings highlight the morphological heterogeneity within *Heterolaophonte* and suggest that none of the three proposed male traits can reliably serve as autapomorphies for the genus. Clarifying the phylogenetic structure of the genus will require integrative approaches combining detailed morphological assessments with molecular data. In particular, efforts should focus on discovering and describing the male morphology of species currently known only from females, as well as testing morphological hypotheses using independent datasets.

### ﻿Ecological implications of colonization on MPD

Floating anthropogenic debris, particularly plastics, has increasingly been recognized as significant vector for the passive dispersal of marine organisms (Gregory 2009; Kiessling et al. 2015; Rech et al. 2016; García-Gómez et al. 2021). Such debris serves not only as transport but also as a secondary substrate for colonization, enabling benthic and fouling organisms—including algae, invertebrates, and small crustaceans—to expand their distribution ranges beyond their natural dispersal capabilities.

*Heterolaophonte discophora* was found on various types of marine debris—specifically a rope, an expanded polystyrene buoy, and a fishing net—all covered with attached macroalgae, including green algae. This, with previous records of the species from habitats associated with coralline or green algae (Song 2000; present study), suggests a strong ecological association with macroalgal substrates. *Heterolaophonte discophora* co-occurred with a variety of harpacticoid copepods across these substrates: the rope hosted species of *Dactylopusia* Norman, 1903, *Sarsamphiascus* Huys, 2009, and *Halectinosoma* Vervoort, 1962; the buoy yielded *Halectinosoma*, *Harpacticus* Milne Edwards, 1840, *Sarsamphiascus*, and *Paramphiascella* Lang, 1944; and the fishing net supported *Ameira* Boeck, 1865, *Paralaophonte* Lang, 1948, *Harpacticus*, and *Diarthrodes* Thomson, 1883. The consistent presence of *H. discophora* on macroalgae-covered debris, regardless of substrate type or associated fauna, highlights its apparent ecological preference for macroalgal habitats. Conversely, other *Heterolaophonte* species shows associations with different invertebrates: *H. brevipes* was discovered among intertidal barnacles, *H. lalanai* from the West Indian fuzzy chiton *Acanthopleura granulata*, and *H. stroemii* from the common shore crab *Carcinus maenas* (Huys 2016).

*Pseudonychocamptus setadefectus* sp. nov. was collected from a gunny sack and an expanded polystyrene buoy, neither of which were covered with macroalgae. This indicates no apparent ecological association with macroalgal substrates, in contrast to *H. discophora*. Among harpacticoid fauna from the MPD, the gunny sack supported a diverse assemblage of copepods, including species of *Dactylopusia*, *Parathalestris* Brady & Robertson, 1873, *Harpacticus*, *Paralaophonte*, and *Sarsamphiascus*, as well as fouling macroinvertebrates such as the Mediterranean mussel, *Mytilus galloprovincialis* Lamarck,1819 and the striped barnacle, *Amphibalanus improvisus* (Darwin, 1854). Conversely, the polystyrene buoy yielded only a few harpacticoid taxa (*Ameira* and *Folioquinpes* Fiers & Rutledge, 1990), with no macroinvertebrates observed on its surface. Although various invertebrates are known to serve as hosts or substrates for certain laophontid species, it remains unclear whether *P. setadefectus* sp. nov. exhibits such an association. These findings suggest that its occurrence on these substrates may instead be facilitated by morphological adaptations common in Laophontidae, such as the strongly developed claw on the P1enp-2 and the robust maxilliped, which enable individuals to grasp fibrous or irregular surfaces (Hicks 1980). For example, *Pseudonychocamptus koreni* (Boeck, 1873) is known to associate with Hydrozoa (*Laomedea flexuosa* Alder, 1857) and Bryozoa (*Bowerbankia imbricata* (Adams, 1798), *Anguinella palmata* van Beneden, 1845) adhering to the host’s substratum (Huys 2016).

In conclusion, the occurrence of laophontid harpacticoid copepods on MPD underscores the potential role of these substrates not only in enhancing the dispersal capacity of benthic species but also in creating novel microhabitats for colonization.

## Supplementary Material

XML Treatment for Pseudonychocamptus setadefectus

XML Treatment for Heterolaophonte discophora

## ﻿Additional information

### Use of AI

No use of AI was reported.

### Author ORCIDs

Kyuhee Cho https://orcid.org/0000-0002-5006-5713

Jong Guk Kim https://orcid.org/0000-0001-5299-9838

Jimin Lee https://orcid.org/0000-0001-9004-8275

### Data availability

All of the data that support the findings of this study are available in the main text.
